# Application of three-dimensional printing in the planning and execution of aortic aneurysm repair

**DOI:** 10.3389/fcvm.2024.1485267

**Published:** 2025-01-29

**Authors:** Harshank Patel, Paul Choi, Jennifer C. Ku, Rosemary Vergara, Rafael Malgor, Dhiren Patel, Yong Li

**Affiliations:** ^1^ Department of Internal Medicine, Western Michigan University Homer Stryker M.D. School of Medicine, Kalamazoo, MI, United States; ^2^ Department of Vascular Surgery, Colorado University, Denver, CO, United States; ^3^ Department of Surgical Sciences, Western Michigan University Homer Stryker M.D. School of Medicine, Kalamazoo, MI, United States; ^4^ Department of Vascular Surgery, Southern Illinois University, Springfield, IL, United States

**Keywords:** endovascular aneurysm repair, 3D phantoms, artificial intelligence, thoracoabdominal aortic aneurysms, physician-modified endovascular grafts

## Abstract

**Introduction:**

The accuracy of fenestrations in stent grafts for complex aortic aneurysms and dissections can be significantly improved using three-dimensional (3D)-printed phantoms. Standardization is enhanced by using artificial intelligence (AI) for image pre-processing before 3D printing. These methods address fallacies in centerline image analysis and manual image pre-processing. This review examines the application of 3D printing and AI in complex aortic aneurysm repair, highlighting current clinical trends.

**Methods:**

An exhaustive literature review was performed using keywords such as “3D printing,” “Artificial intelligence,” “Thoracoabdominal aneurysm,” “Abdominal aortic aneurysm,” “Aortic arch aneurysm,” “Endovascular repair,” and “Open repair” in PubMed and Google Scholar indexes up to June 2022.

**Results:**

This analysis included seven studies: four focused on 3D-printed phantoms for endovascular repair of various aortic pathologies (aortic arch, thoracoabdominal aorta, juxtarenal and pararenal aorta), one on open thoracoabdominal aneurysm repair using 3D-printed models for graft construction, and two on the use of convolutional neural networks, an AI-based technology, for the pre-processing of aortic computed tomography angiography images.

**Conclusion:**

The application of 3D printing and AI-based image pre-processing in the planning of complex aortic aneurysms offers several benefits, including enhanced patient and trainee education, more accurate fenestration placement, reduced surgical time and complications, and decreased surgeon stress.

## 1 Introduction

The endovascular technique to address abdominal aortic aneurysms (AAA) and thoracoabdominal aortic aneurysms (TAAA) has become the norm, with recent developments in techniques and instruments for endovascular aneurysm repair (EVAR) and thoracic endovascular aortic repair (TEVAR), provided that their anatomy is conducive (1). An endovascular repair (ER) has lower perioperative morbidity and mortality when compared with open surgical repair, which is an extensive procedure, with operative mortality reported to be 9.5%, even in expert hands (2–4). Thus, an ER is particularly favored in patients who are too frail for an open surgical repair (2, 3). There are essentially three modalities of ER for complex aortic aneurysms: a parallel technique with snorkel/chimney grafts, company-modified devices (CMD), and physician-modified endovascular grafts (PMEG), although the latter two are not always applicable to cases with hostile anatomies (5, 6).

CMDs require up to 8 weeks before delivery and are unlikely to be fitted for those requiring urgent intervention. Moreover, this latency is associated with a reported mortality rate of 4% (7, 8). CMDs by Cook (Cook Medical, Bloomington, IN, USA) and Terumo (Terumo, Inchinnan, UK) tailored to individual patients cost over $15,000 in retail, not including the adjuncts (9). On the other hand, though parallel techniques with snorkel/chimney grafts can be employed in instances where the anatomy of the disease is unfavorable, they elevate the risk of type Ia endoleak up to 13.4%, and this complication may not be correctable and may even be lethal if it ruptures (10). Thus, PMEGs are a commonly favored modality, especially if urgent intervention is needed.

Using a PMEG mandates meticulous planning with the employment of advanced and precision-centered radiological interpretation of the diseased aorta and accurately locating the fenestration sites (11). This often relies on the centerline of the aortic flow, which could be misleading as it does not consider the interplay between the endovascular graft and the tortuosity of the native aorta, which could alter the relationship between the fenestrations and ostia for visceral and segmental vessels (12). Moreover, this technique is susceptible to inter-observer variability (13). In addition, varied modification techniques are employed at different centers, contributing to the lack of standardization (14, 15). Furthermore, there are no quality control means for PMEG (7, 8). Other points of concern are the prolonged time required to create the fenestrations on the endograft and likely unreliable placement.

These constraints can be aptly addressed by three-dimensional (3D) printing technology, in which a specific material is added layer by layer to formulate the desired shape with precision (16, 17). In the field of vascular surgery specifically, renditions of imaging such as computed tomography (CT) angiograms, followed by post-processing with software like Mimics (Materialise, Leuven, Belgium) and 3D Slicer (Harvard, Boston, MA, USA), aid in obtaining 3D-printed aortic “phantoms.” These phantoms are anatomically far more superior than imaging alone, with accurate positioning of the branches, nuanced appreciation of the diseased aorta, and physical probing, thus allowing for seamless synergism between the surgeons and the radiologists to develop the most appropriate action plan (18, 19). Moreover, these models can be used to curb the fallacies of centerline analysis, namely inter-observer variability and shuttering phenomenon (13). The latter takes place when the fenestration fails to coincide precisely with the native vessel's ostium (20). Furthermore, the endovascular graft can be deployed within the 3D-printed phantom *in vitro*, which emulates the interactions between the native aorta and the graft *in vivo*, leading to a more accurate fenestration placement for the diseased aorta. Moreover, the phantom aids in identifying strut-free zones on the graft for fenestration placement (13, 20, 21). Marone et al. also postulated that these 3D-printed models, when used in preoperative planning for complex aortic aneurysms, can lead to decreased surgical time and complications by curbing futile efforts during the procedure and aiding in ascertaining the exact materials most appropriate for the procedure (22). They are not only used for patients but also in trainee education (23–29).

The principles of 3D printing can also be applied to open TAAA repair. An open repair carries an inherent risk of paraplegia, especially in the case of Crawford type II and III aneurysms (Crawford criteria refers to the anatomical classification of TAAA based upon the extent of aortic involvement). Spinal cord perfusion is heavily dependent on the segmental vessels at the levels between T8 and L2. Thus, large intercostal vessels are normally included in TAAA repair (4, 30–33). This is either done by including a single patch with origins of the said vessels in the Crawford “island/inclusion patch” repair or, more preferably, by the so-called “octopod” technique. In this technique, a multibranched aortic graft is pre-sewn. This is especially preferred in patients with connective tissue disorders, such as Marfan syndrome, which could lead to aneurysmal degeneration of the patch itself (34–36). Rhee et al. carried out a small comparative analysis of open TAAA repair with this technique with or without the aid of 3D-printed models in the manufacturing of the octopod graft (35). They marked the visceral and segmental branches as small protrusions on the model. They found that the 3D-printing technology led to greater procedural efficiency and favorable early and mid-term outcomes. They also concurred that 3D-printed models led to quicker recognition of appropriate contenders for segmental vessel revascularization, quicker anastomosis between the grafts, tailoring appropriate arrangement of the graft even in cases with hostile anatomies, decreased surgeons’ stress, a greater number of segmental vessel anastomosis, and potentially greater patency rates due to beneficial hemodynamics owing to the decreased length and angulations in graft configuration (35).

The aim of the present article was to present the basics of 3D printing and analyze the effects of the current trends in its applications in planning the repair of complex aortic aneurysms. It also aims to present a brief analysis of the application of Artificial Intelligence (AI) in the context of 3D printing for vascular surgery.

## 2 Methodology and application of AI in 3D printing

### 2.1 The basics of 3D printing

For 3D printing, images can be acquired via multiple modalities, such as CT, magnetic resonance imaging (MRI), or even ultrasonography (USG), and are stored in the Digital Imaging and Communications in Medicine images (DICOM) format (37, 38). CT is the most frequently used modality as its images carry a variety of applications and can be easily post-processed (17). In these images, different tissues should be distinguished and of high enough resolution, lest there be significant differences between the phantom tailored and the native anatomy (2, 9, 17). Moreover, the image should be sliced into less than 1 mm (16).

This image is now subject to segmentation, also known as labeling (because pixels, if it is a 2D image, and voxels, if it is a 3D image, are allocated a different label and share a particular label if they share similar features) (39). Then, the images are sliced into different anatomic regions of interest (ROI) (40). Segmentation can be both manual and automated, with the former being an extremely time-consuming and laborious process (17, 41). Moreover, if it is done manually, there is room for inter-observer variability as it requires a thorough understanding of the anatomy so that only ROIs are selected, and no tissue from outside the ROIs is included (42). Segmentation is also pivotal in determining the eventual accuracy of the 3D rendition (16).

After segmentation, the image is subjected to another process called mesh generation. Here, the voxels of image segments are transformed into a mesh consisting of triangular facets with automated software. This leads to a rendition with a smoother surface but is a close estimate of the original image (43). Hence, supplementary corrections of artifacts are required, either manually or by using automated software (44).

The post-processed DICOM image must then be converted to a format recognizable by a 3D printer. This most commonly can be in the Standard Tessellation Language (STL) format. Herein, individual surfaces of the post-processed image are defined in the form of triangular facets that fit together into a cohesive structure. This format is recognizable by a 3D printer instead of the entire DICOM image, which a printer cannot recognize. By recognizing the ROIs in the STL image as 2D structures bound by successive cross sections, a 3D printer lays a specific material in respective layers, which are then fused to yield the desired structure (3). One must note that the resolution of a 3D printer is superior to that of radiological images obtained. Thus, various corrections are required, including topological correction, decimation, Laplacian smoothing, and local smoothing (45–48). When the image is finally ready, it is transferred to a 3D printer. There are a variety of 3D printers working with different principles and mechanisms.

### 2.2 The types of 3D printers used in the field of vascular surgery

Stereolithography (SLA) printers fabricate 3D structures using photosensitive liquid-based resins. The resin is layered, followed by precision-based ultraviolet (UV) irradiation, guided by computer-aided design (CAD) files. This leads to the generation of free radical species from the resin, polymerizing it into a solid structure. The initial layer formed is attached to a supporting platform, which moves in a predefined fashion. Each sequential layer is particularly placed as programmed and irradiated to yield the desired 3D structure (49). PolyJet printers are another class of printers. Herein, drops of photopolymer constitute a layer, which is then UV irradiated. This process is repeated until the desired structure is achieved. Support materials with a gel-like consistency can be customized to reinforce the 3D structure and can be later removed manually or by water jetting (50). On the other hand, the photopolymer could constitute an admixture of support and core building materials that are irradiated after each layer is placed. PolyJet printers are versatile and can utilize an array of different materials (51). Conversely, a single structure made of multiple materials can be fabricated (51). A plastic polymer filament is used in the fused deposition model (FDM) technique. As the filament is brought into contact with a heating head, it transforms into a soft, semi-solid state and then is thinly layered on a bed. Now, with the maneuvering of the bed or the heating pad, the next layer is lined up at predefined 3D coordinates to construct the desired 3D object with accurate dimensions. Two consecutive layers are bound to each other by heat-induced molecular interactions (52). Noticeably, objects fabricated with the help of FDM may require support structures (53). Multi-jet printing (MJP) works on a principle very similar to PolyJet. A transparent acrylic photopolymer is used as the corebuilt material, and wax as a support material. Both of these are dispersed by the printer head together on a tray, followed by UV irradiation. The tray moves to precise coordinates for each layer to be laid at the desired location, manufacturing the required 3D object. The drawback of this method is that the acrylic photopolymer is relatively weak and is prone to contortion at angulations of 65° or greater (16).

After the 3D model is printed, it is usually imperative to sterilize it, especially if it must directly contact a patient's surgical field or any graft or implant meant for a patient. The modalities for the same may include high temperature (e.g., steam, flash autoclave), chemicals (e.g., ethylene oxide, hydrogen peroxide, peracetic acid), and radiation (54). Typically, 3D-printed objects favor the latter two modalities, such as ethylene oxide or gamma radiation (55).

As mentioned earlier, the segmentation process may be automated and thus effectively curbs the strictures of manual segmentation. This can be achieved with the application of AI. Essentially, AI refers to a computational program that can carry out functions that usually require human intelligence. This may include recognizing and identifying a pattern, planning, language comprehension, object and sound detection, and problem-solving (56). In terms of its application, AI can be characterized as possessing a machine-like capability, as it makes automatic and unaided decisions on the grounds of the data fed to it (56). Thus, AI analyzes sizable data, detects patterns, and ascertains possible results at its crux.

### 2.3 The basics of AI

The basic functioning principles of AI include the following four notions: machine learning (ML), artificial neural networks (ANN), natural language processing (NLP), and computer vision (CV) (57).

ML pivots on the process by which computers learn from the fed data (58, 59). The working algorithms of ML can be supervised, unsupervised, or semi-supervised. This classification is based on the extent of inclusion of the outcome of interest into the algorithm to prime it to obtain a formidable predictive power (60). Supervised mode is used when it is desirable to prime an ML algorithm, which ensures a known outcome with high fidelity. On the other hand, the unsupervised mode is used to ascertain a tangible and previously unrecognized pattern from a large set of data (61). ML is especially fitted to recognize elusive patterns in extensive datasets, which are usually not readily perceptible to manual interrogation (62). NLP constitutes a multidisciplinary approach wherein AI, computer science, and linguistics intersect to empower machines to comprehend human languages to yield consequential results (57). ANN functions on the fundamentals of its biological counterpart, in which each neuron tends to be intertwined with other neurons (63). ANN constitutes an input layer, wherein datasets are introduced, a hidden layer that processes the data, and an output layer that releases the final results (64). Deep neural networks work on a similar principle but consist of multiple layers and thus can elucidate more intricate and elusive patterns than their simpler, uni- or bi-layered counterparts (65). CV simply means the analysis of images and videos with human-like insight. In medicine specifically, CV has utility in image acquisition and interpretation in axial imaging, leading to a computer-aided diagnosis, image-guided surgery, and virtual colonoscopy (66).

Now, utilizing the above principles of AI, segmentation can be automated, leading to decreased time to complete analyses, alleviating the burden of tedious labor by the radiologist, and improving reproducibility. This is especially relevant as the process of segmenting images obtained for vascular intervention is particularly intricate, owing to inconsistent and variable anatomy of the vessels and aortic aneurysms. Furthermore, associated atherosclerotic occlusive disease, calcification, intramural thrombus, previous stent grafts, tortuosity, and anatomic relation with the adjacent structures render this process even more demanding. Moreover, disturbances may arise due to technicalities entailed in imaging modality, contrasts used, resolution of the obtained images, and potential artifacts and noises. ML algorithms for aiding in analyzing aortic aneurysm images are under the pipeline (67). This technology should improve image acquisition, measurements, interpretive reporting, and the associated costs (68). Table 1 displays an array of such technology and its applications for planning endovascular aortic repairs.

**Table 1:** An overview of 3D-printed phantom use in PMEG construction for aortic aneurysm and dissection repair.

Source	Tong et al. (*n* = 34) (69)	Branzan et al. (*n* = 12) (70)	Rynio et al. (*n* = 43) (71)	Tong et al. (*n* = 17) (72)
Pathology addressed	Thoracoabdominal aneurysm and dissections	Thoracoabdominal and pararenal aortic aneurysm	Thoracoabdominal, juxtarenal, suprarenal aortic aneurysms	Aortic arch aneurysms and dissection
Image analysis software	Mimics software (version 21.0; Materialise, Leuven, Belgium)	Geomagic DesignX	3D Slicer 11.0	Mimics software (version 21.0; Materialise, Leuven, Belgium)
3D printer	Eden260VS (Stratasys, Inc., Gilbert, AZ, USA)	Stereolithography 3D printer Form 2 (Formlabs; Somerville, MA, USA)	Form 2 printer (Formlabs, Somerville, MA, USA)	Eden260VS (Stratasys, Inc., Gilbert, AZ, USA)
Phantom manufacturing time (h)	5	7	6 ± 1.9	5
PMEG creation time (h)	1.26 ± 0.35	1.83 ± 0.18	1.43 ± 0.19	1.25 ± 0.46
Sterilization mode	Ethylene oxide gas	Steam pressure	Hydrogen peroxide plasma or ethylene oxide gas	Ethylene oxide gas
Total operative time (h)	5.62 ± 1.25	2.68 (range 1.32–4.48)	4.12 ± 1.16	4.18 ± 1.57
Endovascular intervention time (h)	2.84 ± 0.54	1.3 ± 0.48	Not disclosed	2.07 ± 0.54
Fluoroscopy parameters	Contrast volume (ml): 224.58 ± 45.33 Radiation dose (mGy): 2,885.45 ± 487.56	Fluoroscopy time (min): 55 (range 17–99) Contrast volume (ml): 77.7 ± 34.9	Contrast volume (ml): 217.67 ± 36.70	Contrast volume (ml): 225.45 ± 46.13 Radiation dose (mGy): 2,731.52 ± 550.62
Hospital stay (days)	10.22 ± 3.65	17.3 (range 5–38)	8.06 ± 12.49	8.72 ± 2.93
Intensive care unit (ICU) admission duration (days)	0.82	2.8 (range 1–14)	Not disclosed	0.65 (range−0–3)
Number of fenestrations	107 fenestrations [102 bridging stent grafts (73 covered stents and 29 bare stents)]	71 fenestrations	162 fenestrations (mean of 3.8 per patient)	Triple pre-fenestrated stent graft: 12 patients Double pre-fenestrated stent graft + single *in situ* fenestrated stent graft: 3 patients Single pre-fenestrated stent graft + double *in situ* fenestrated stent graft: 2 patients
Morbidity	Minor cerebral infarct (1); endoleaks (5; 4 required intervention)	Transient spinal cord ischemia with full recovery (2); acute kidney injury (AKI) with partial recovery (1); endovascular reinterventions (2); endoleaks (3)	Paraplegia (1); Stroke (1); AKI not requiring permanent dialysis (3); ICU stay (8); endoleaks (12)	Endoleak (1)
Mortality	1 (ruptured retrograde dissection)	1 (sepsis due to *Mycobacterium bovis* BCG after 1 year of endovascular repair)	30-day mortality: 5 (1: iliac artery rupture; 1: arrhythmia; 3: multisystem organ failure)	None

## 3 Current experiences with AI-driven 3D printing in aneurysm repair

The recent experience of the utility of 3D printing in TAAA repair is mainly reported in technical notes, case series, single-center experience, and small prospective cohort studies. Case reports with single patient experience and studies with only an abstract without the full manuscript were excluded. Studies that reported, in sufficient detail, regarding image analysis, phantom creation, operative steps, as well as branches fenestrated, fluoroscopy parameters, complications, morbidity and mortality outcomes, and with a minimum mean postoperative follow-up period of at least 6 months were included. Finally, a single study that reported their experience with the use of 3D-printed phantoms in open aortic aneurysm repair was included, as it directly compared a limited set of outcomes with conventional repair.

In 2020, Tong et al. reported treating a total of 34 patients with either TAAA (*n* = 15) or thoracoabdominal dissection (*n* = 19) treated with PMEG constructed with the help of a 3D-printed phantom at their institute (69). Computed tomography angiography (CTA) images with a slice thickness of 0.625 mm were obtained. Stent grafts used were Ankura (Lifetech Scientific Corporation, Shenzhen, China), Zenith (Cook Medical, Bloomington, IN, USA), or Endurant (Medtronic, Minneapolis, MN, USA) with a variety of balloon-expandable or self-expanding bare metal bridging stent grafts. The main aortic stent graft was fully deployed within the printed phantom, and each fenestration was marked and created using an electric pen (CIRX Inc., Ningbo, China). The diameter of the fenestrations located in the diseased segment was approximately 5–8 mm, which was usually smaller than the branch to prevent endoleaks. The diameter was 10 mm if the fenestration was located within the non-diseased vessel. They were reinforced by suturing radiopaque wires with non-absorbable sutures. A covered branch attached to the main body with fenestration of the size of the actual arterial branch was tailored in cases of a true aneurysm, in which there was a large space between the stent graft and sac wall. With two lower-limb and one left upper-limb accesses, the two renal arteries (RA) and superior mesenteric artery (SMA) were first secured, followed by insertion and release of the aortic stent graft. Each arterial branch was cannulated through its fenestrations. Two target branches were lost during the procedure in aortic dissection patients due to their involvement in the dissection itself; however, they were successfully addressed in the second stage of the procedure. One death (3%) was observed 1 week postoperatively due to ruptured retrograde dissection. No renal failure or paraplegia was observed. The mean follow-up time was 8.5 months.

At their facility, Tong et al. reported no significant adverse events in the short term with the conventional chimney stent-graft method; the incidence of endoleaks and bridge stent grafts was significant in the long-term follow-up. With the employment of the 3D-printing––aided PMEG, the incidence of these events on long-term follow-up is unknown, and the incidence of endoleaks at the first postoperative CT scan was significant (*n* = 5, 14.7%). Furthermore, there could be a purported benefit of a more physiological blood flow with this novel technique due to the lack of a large space between the main aortic stent graft and the branch chimney stent grafts, reporting of the outcomes with longer follow-up times is required to support this hypothesis.

In 2021, Branzan et al. described a series of 12 high-risk patients with symptomatic or contained rupture of Crawford type IV TAAA (*n* = 7) or pararenal aneurysms (PRA) (*n* = 5) treated with 3D-printing–assisted PMEG insertion (preceded by seven other patients whose procedures were planned using 3D image technology) (70). All patients were either hemodynamically stable on presentation or were stabilized medically before the procedures, providing enough time for 3D printing of the phantoms. Valiant Captivia Closed Web (Medtronic, USA) was used for all but one patient. Fenestrations were marked with a sterile pen after deployment of the graft within the phantom, followed by the creation of the fenestrations to get the necessary 20 mm seals using a surgical scalpel, and they were then reinforced. The PMEGs were soaked in a rifampicin solution.

A single surgeon performed the procedure, and the technical success rate was 100%. Thus, all 71 fenestrations created yielded revascularization. No perioperative mortality or conversion to open repair was observed. The mean follow-up time was 14.4 months, during which one mortality from *Mycobacterium bovis* (BCG) sepsis was observed, but no aneurysm-related mortality was noted. The branch occlusion rate was 5.4%, and the all-cause reintervention rate was 10.6%. The mean PMEG manufacturing time was decreased with the employment of 3D-printed phantoms. This was partly due to printer performance and improved surgeon's experience.

Branzan et al. reported the experiences of a single operator only over a period of 4 years, possibly indicating a steep learning curve. In addition, seven patients were treated with 3D image analysis software-guided PMEG construction before the employment of 3D-printed phantom grafts, which could be contributing to a greater level of comfort with this novel technique. Furthermore, most patients either had type IV thoracoabdominal or juxtarenal aortic aneurysm, unlike in the study by Tong et al, which had a more balanced distribution of thoracoabdominal aortic disease types.

In 2022, Rynio et al. similarly reported a single-center study with patients who did not have prior experience with fenestrated endovascular aneurysm repair (FEVAR) or branched endovascular aneurysm repair (BEVAR) (71). They treated 43 patients with juxtarenal and suprarenal aortic aneurysms, type IV TAAA, and type IA endoleak after endovascular aortic repair. A single vascular surgeon segmented all of the CTA images. Fenestrations were created after deploying the stent graft in the printed phantom for the two RA, SMA, and celiac artery (CA) for all patients whenever they were patent. The SMA fenestration was preloaded with a soft guidewire before repacking it into the insertion system. In all cases, the proximal deployment was in zone 5 (i.e., above the CA). The two-stage procedure was carried out in 18 (42%) patients who were asymptomatic with aortoiliac stent grafts to avoid paraplegia.

The cost of the phantom was US$5 ± 2. Valiant Captiva (Medtronic, Dublin, Ireland) was used as the PMEG in all patients. Of them, 29 (67%) required a distal stent-graft component. The technical success rate, i.e., successful PMEG deployment with cannulation of the target vessels and no death within 24 h, was 86%. Two patients died within the first postoperative day due to iliac artery rupture and arrhythmia, respectively. Another three patients died within the first 30 postoperative days due to multisystem organ failure. One patient died during the hospitalization due to pulmonitis and paraplegia. Total in-hospital mortality was 14%. The mean patient follow-up was 14 ± 12 months and the total mortality rate was 40% (*n* = 17) at the end of this period. During follow-up, 95% of SMAs, 93% of right RAs (occlusion of one was due to cannulation failure in the index procedure), 95% of left RAs, and 89% of CAs remained patent. Beyond this period, one procedure-related death was noted and was due to acute thrombosis of three bridging stents 540 days after the index procedure. A total of 10 additional deaths occurred due to gastrointestinal bleeding (*n* = 1), general surgical complications (*n* = 1), acute pancreatitis (*n* = 1), pulmonitis (*n* = 1), malignancy (*n* = 2), and from unknown causes (*n* = 4). The overall procedure-related mortality was 16.27%. The reintervention rate was 19% and mainly represented high-pressure endoleaks (*n* = 12, 28%), but one was due to acute thrombosis of the SMA and both renal stents. Two reinterventions were required in the early postoperative period due to rupture of the iliac artery and closure of the SMA stent. There was no statistical significance among symptomatic and asymptomatic patients concerning 30-day mortality, in-hospital mortality, and technical success. It should be noted that their follow-up period had significant variability (14 ± 12 months); hence, complications such as endoleaks and loss of patency of any of the cannulated vessels in the later periods may not be reliably accounted for.

In 2019, Tong et al. performed an endovascular repair of the aortic arch of 17 patients with the help of 3D printing (72). Five patients had TAAAs and 12 had aortic arch dissections. Ankura and Captiva thoracic aortic stents were used. Fenestrations were created with an electric pen (CIRX Inc., Ningbo, China) and the edges were trimmed with microscissors after deploying the stent graft in the phantom and were reinforced. Their diameter was a little smaller than that of the branch stent to reduce the risk of endoleaks. The fenestration diameter was mainly 5–7 mm; it was sometimes over 10 mm when no laceration was present in the dissected arch. A 10–20 mm wire was sewn into the fenestration when a large gap was present between the arch wall and the stent. A radiopaque wire was sewn on the greater curvature of the graft and a 0.018-in. guidewire on the lesser curvature to prevent potential fenestration misalignment from torsion while placing a stent delivery sheath and to reduce the diameter of the anterior segment, respectively. The constrained diameter ensured blood flow to the arch branches while cannulated perioperatively, reducing the risk of stroke and obviating the need for any additional bypass procedure. On the other hand, the constraining wire did not hinder the release of the fenestrations and branches because it was placed opposite to them. Covered stents were used for most branches, followed by balloon expansion, further reducing the risk of endoleaks.

Of the 17 patients planned for PMEG, 16 were treated as above, while one was treated with a chimney stent with *in situ* fenestration. The technical success rate was 94.18%. The mean follow-up was 6 months (range 3–14 months). Follow-up CTA at 3 and 6 months revealed no obstruction of the three arch branches; the aneurysm or dissection was thrombosed without any progression. One patient had a small endoleak related to a bare metal stent in an innominate artery, which was managed conservatively. Fenestration misalignment occurred in the second patient in this series and was attributed to the learning curve associated with this novel technique and hostile arch anatomy. No mortality or other complications were observed. Notably, the success rate for this study, by extending the use of 3D-printed phantoms to endovascular aortic arch repair, potentially indicates the versatility of this approach, with applications to other vascular areas as well. But the outcomes that they achieved would be difficult to replicate, as their center had extensive working experience with this method, as reported earlier (69).

Rhee et al. demonstrated the utility of 3D printing in open TAAA repair. They performed a retrospective analysis of open TAAA repair performed by a single surgeon at a center where this procedure was performed regularly (35). Then they compared and contrasted the cases where 3D printing was used for preoperative planning with a control group. In total, 20 patients with Crawford II or III TAAA due to either a degenerative disease or dissection and who were treated with the octopod technique were included in the study. Repair in nine patients was supplemented with 3D-printing technology, while the other 11 patients did not receive this treatment. For the former group, electrocardiography (EKG)-gated CT images were obtained for centerline analysis and segmentation. Two types of phantoms were printed: one was a visualization guide, wherein the grafts for the segmental and visceral branches were attached to the main aortic graft, and the other was a marking guide, which had protrusions at the site of these branches to aid in marking fenestrations on the main graft. VisiJet PXL Core powder, VisiJet PXL clear binder, and Color bonds were used to print them. A four-branched TAAA Coselli thoracoabdominal graft (Vascutek Ltd., Renfrewshire, UK) was then deployed into the sterile phantom. Then, with the aid of the protrusions, segmental branches were marked, and 10 mm Hemashield Platinum straight grafts (MAQUET Cardiovascular LLC, San Jose, CA, USA) were then sewn there. However, for visceral vessels, the four-branched TAAA graft was used as such, with CTA guidance but without input from a 3D-printed phantom due to perceived homogeneity in the visceral branch's origin. The type of image processing software, phantom manufacturing time and cost, PMEG creation time, and sterilization method for the phantom were not disclosed.

Eight patients (72.7%) in the non-3D-printing guided (n3D) group and five (55.6%) patients in the 3D-printing guided (3DG) group had Marfan syndrome. Five patients (45.5%) in the n3D group required a bi-iliac reconstruction distally, unlike patients in the 3DG group. A mean of two (range 1–3) segmental arteries per patient in the n3D group and three (range 0–4) segmental arteries in the 3DG group were anastomosed. Different operative times in both groups are summarized in Table 2.

**Table 2:** A comparison of different operative times and lengths of hospital stay in Rhee et al.’s series of open thoracoabdominal repair between groups with and without the guidance of 3D-printed phantoms (35).

Operative times	Non-3D printing guided group	3D printing guided group
Cardiopulmonary bypass time (min)	181 (IQR: 148.5–198)	170 (IQR: 124–175)
Total circulatory arrest time (min)	13.5 (IQR: 10.8–14.5)	12 (IQR: 11–13)
Total operative time (min)	437 (IQR: 413.5–475.5)	489 (IQR: 414–496)
Hospitalization duration (days)	4 (IQR: 3–6)	6 (IQR: 5–10)
Intensive care unit admission duration (days)	16 (IQR: 14.5–25.5)	22 (IQR: 15–29)

No perioperative mortality, 30-day mortality, or postoperative stroke was observed. Complications in the n3D group included one patient who developed low cardiac output syndrome and required veno-arterial extracorporeal membrane oxygenation (VA-ECMO) for 45 h before weaning and three patients who required new-onset temporary dialysis. One patient in the 3DG group developed permanent paraplegia. This patient did not have Marfan syndrome but had undergone Crawford I TAAA repair with three pairs of intercostal vessels as part of the island patch repair; during the repeat Crawford III TAAA repair, one of the two segmental arteries targeted for revascularization was damaged during adhesiolysis. Two patients required surgical re-exploration, one from each group, and six patients required prolonged ventilatory support, three from each group.

There are multiple methods by which AI can be applied in the automated segmentation of CTA images for aortic aneurysm repair planning. Saitta et al. utilized convolutional neural networks (CNN), a multilayer neural network that requires fewer input data than its conventional counterpart and hence is easier to train and is often more productive. It creates deep learning (an ML subtype based on a multilayered neural network where several layers are hidden) based pipeline (a series of processing entities that yields an output that acts as an input for the subsequent processing unit) for geometric analysis for TEVAR planning (73).

Saitta et al. manually segmented the thoracic aorta (up to the CA) and pulmonary arteries from 465 CT scans. Of these, 9 patients had TAAAs, and 219 patients had a common origin of the innominate and left carotid arteries (CILCA) arch. This set was then divided into two groups: scans of 395 randomly selected healthy patients and ground truth segmentations to be fed to CNN for training, and 70 scans (including 9 TAAA cases) for the test group. CILCA scans were present in both groups. A 3D U-Net architecture was used to train the CNN to yield a fully segmented thoracic aortic image, aortic arch centerline radius of curvature, proximal landing zones (PLZ), and their maximum diameters, angulations, and tortuosity. The images were segregated into patches of 128 × 128 × 128 pixels, and the said patches were divided into two groups (or batches) to be separately processed (74).

The trained CNN then analyzed the data, and the results were expressed concerning the parameters above. They were then compared between the scans with standard aortic arches and ones with CILCA arches. Its performance was quantified by comparing its result with manual ground truth segmentation values using Dice coefficients (DC) and Hausdorff distances (75). They were 0.954 (range 0.873–0.999) and 11.97 (1.96–68.83) mm, respectively. Moreover, the CNN could correctly identify and characterize the nine TAAA cases. Furthermore, there was a statistically significant difference between both arches with respect to maximum zone diameters (*p* < 0.0001), angulation (*p* < 0.0001), and tortuosity (*p* < 0.0001). In addition, the angulations and tortuosity in PLZ3 of CILCA arches were significantly greater (*p* = 0.015 and *p* = 0.048, respectively) than in the standard arches. This CNN required less than 7 min to process one image (approximately 4 min for segmentation and 3 min for geometric analysis), and this was much shorter than that of the manual segmentation employing commercially available software, which needs approximately 30 min a case.

Similarly, Fantazzini et al. (76) trained a CNN model to automatically segment the entire aortic segment, i.e., from the ascending aorta to the iliac arteries. Their deep learning-based pipeline also accounted for 3D spatial nuances. First, with the help of a 2D U-Net architecture, the CNN trained on axial slices reduced to a quarter of the original to coarsely characterize the aorta from the entire CTA image, followed by processing of the same coarse aorta in x, y, and z axes to obtain a segmented image in axial, sagittal, and coronal planes, respectively, under higher resolution, and integration of these orthogonal image planes to get a final 3D segmented image that obeys triplanar spatial nuances. This methodology curbs the shortcomings of single-plane CNNs.

In total, 80 postoperative CTA images of AAA, including thoracic, abdominal, and iliac portions, were included after semi-automatic segmentation with the help of the ITK-Snap tool (77). Ground truth segmentations of 64 scans were used to train the CNN, 6 scans were used for validation, and 10 were used to test the network. The validation involved regularization to curb data overfitting, determining the threshold for binarizing the crude probability maps provided by the network and ascertaining the most appropriate method for integrating the three uniplanar predictions (77).

The DC for the coarse preliminary segmented aortic image was 0.92 ± 0.01. Herein, Jaccard scores and surface-to-surface distances were not considered, given the nature of the image. Similarly, the DC for individual planar images i.e., axial, sagittal, and coronal sections, were 0.92 ± 0.02, 0.92 ± 0.04, and 0.91 ± 0.02, respectively. Finally, three modes were considered for integrating these images: simple averaging, majority voting, and a combination of the two. The validation set assessed these modes, and it was found that all of the approaches yielded similar results. Nonetheless, the combination method was chosen due to slightly better results. The DC was 0.930 ± 0.021, the Jaccard coefficient was 0.870 ± 0.036, the mean surface distance was 0.559 ± 0.188 mm, and the maximum length was 28.020 ± 5.807 mm. When compared with the results of individual planar images, it was revealed that the integration of the segmented orthogonal images yielded better results. Overall, it took approximately 150 h to manually generate the dataset consisting of ground truth segmented images, approximately 18 h for training the networks, and only 25 ± 1 h for the final automated segmented image generation by the trained pipeline.

## 4 Future directions

PMEGs are a safe and effective method for acutely symptomatic or contained rupture of an aortic aneurysm (78). The time required for constructing a PMEG dictates the expeditiousness of treatment. When an aortic phantom is used to improve the accuracy within a PMEG, one must also consider the time required for image processing and 3D printing. Thus, the total time may take up to several hours, as seen in various previous studies; however, this is much shorter than the several weeks it takes to order a CMD (7, 69–72). Similarly, 3D-printed phantoms have improved the accuracy and specificity of the octopod grafts for open TAAA repair, with decreased operative times due to simplified and accelerated anastomoses with segmental vessels (35). The reduced time also decreases visceral ischemia time (69). Furthermore, with the application of AI in automated image processing and segmentation for 3D printing, the time and effort required have been significantly reduced (79). Yet, the current literature is lacking in marrying these two notions, owing partly to the novelty of these topics.

Herein, note should be taken of the rapid leaps and strides taken by AI and its application, specifically in the field of aortic surgery. Image segmentation algorithms have now been approved by the U.S. Food and Drug Administration (80). This is a major step forward, especially because this approval paves a path for bringing more AI-driven technologies into clinical practice, including image analysis algorithms to improve the accuracy and timeliness of diagnosis of aortic pathologies, such as aneurysm, dissection, and plaque burden (81, 82). With the addition of CNN, these algorithms have also been successfully applied to predicting aneurysm sac expansion (81). Caradu at al. (83) directly compared automatic with manual image segmentation and found a statistically significant correlation between the results of various aortic aneurysm parameters and blood flow characteristics obtained through either method, signifying that these algorithms could act as an important adjunct in clinical practice. ML algorithms have also been used in perioperative planning, including the development of non-radiation electromagnetic-based navigation systems for anatomical mapping and 3D visualization during endovascular aortic repair (84). This could potentially decrease the fluoroscopy time for physicians and patients. Lastly, virtual reality can be extremely useful for trainee education (85).

Rynio et al. revealed that the mean cost of their phantom was US$5 ± 2 (35). Thus, with a one-time investment in a 3D printer, the associated software adjuncts, and the help of AI-trained networks for quicker and cheaper image processing, an urgent repair of complex aortic aneurysms and dissections can be performed in centers without much experience or CMD availability. Thus, 3D printing and AI-driven image processing technologies can decentralize endovascular and open repair of urgent and complex aortic pathologies beyond academic tertiary healthcare centers. Moreover, the task of image analysis and 3D printing of the phantom can be delegated to a separate, discrete center, which can cater to a multitude of institutes while gaining expertise in these techniques and saving costs for smaller centers.

Nonetheless, there are potential restrictions in applying these technologies in real-world settings. Rynio et al. employed the 3D-printing technology in PMEG endovascular repair of aortic aneurysms of various anatomies. Their center had no prior experience in addressing complex aortic cases. Their outcomes reflected that this approach was associated with worse results than those previously reported. This was attributed to the learning curve for F/BEVAR for complex aortic cases rather than to the use of 3D-printed phantoms (35). Further, the materials currently used for 3D printing are rigid and may fail to conform precisely to a tortuous aorta. Thus, for more patients to take advantage of this technology, more flexible and biocompatible materials will need to be developed (70). Moreover, the topic of the vascular utility of 3D printing still lacks randomized control trials (RCT) and systematic reviews. Although results have been favorable in most cases, studies with extended follow-up periods are needed to assess its long-term outcomes. In addition, studies with larger sample sizes are required to improve the quality of the evidence.

Similarly, training AI networks is a data-intensive project. This shall require a standardization of various forms of data reported and the sharing of pertinent patient information across multiple centers over the same platform. This is impossible without a significant overhaul in medical informatics and ethical and legal impediments to patient privacy. Moreover, investments to augment computational prowess would be necessary, though there is a possibility that they may improve patient care and healthcare costs in the long run. Lastly, there will be concerns about a machine replacing a physician's job or drastically affecting the patient–physician relationship. However, it must be noted that AI is a complement and not a substitute for a physician's clinical acumen in patient care.

Finally, 3D bioprinting, the discipline that deals with the fabrication of 3D cell scaffolds and medical implants, represents an even more novel yet exciting avenue. It represents the pinnacle of personalized medicine, in which the very cells, proteins, drugs, and/or other biologically active molecules can be arranged with remarkable spatial and temporal precision and specificity to steer tissue formation (86, 87). As of now, the primary technical stricture lies in finding a biomaterial and bioink with appropriate mechanical and biological properties to mimic the native tissue (88). Only time will tell what lies in the future of this technology.

## 5 Conclusions

3D printing represents a technology that has the potential to uniformize the practice of PMEG for complex aortic aneurysm repair. It may also shorten the physician learning curve by determining the graft's fenestration sites by itself, thus reducing the disadvantages associated with inter-observer variability and making the entire process more precise. The process can be further refined by introducing automated image segmentation to replace its manual counterpart, further streamlining it. Therefore, it is not unrealistic that the repair of complex aortic aneurysms can be tamed with the help of AI-guided image processing and 3D-printed phantoms for accurate fenestration creation, even at centers with little to no prior experience with complex F/BEVAR. By combining the ever-evolving 3D printing and AI technologies, the repair of complex aortic aneurysms can be simplified, expanded, and standardized across centers and even the globe.
